# Association between Rheumatoid Arthritis and Poor Self-Perceived Oral Health in Korean Adults

**DOI:** 10.3390/healthcare10030427

**Published:** 2022-02-24

**Authors:** Hana Shim, Jungwan Koo, Joonho Ahn

**Affiliations:** 1 Department of Public Health, Graduate School, The Catholic University of Korea, Seoul 06591, Korea; shn0412@naver.com; 2 Department of Occupational and Environmental Medicine, Seoul St. Mary’s Hospital, College of Medicine, The Catholic University of Korea, Seoul 06591, Korea

**Keywords:** arthritis, rheumatoid, oral health, cross-sectional studies

## Abstract

Background and objective: Rheumatoid arthritis (RA) and oral health problems have been reported as specific disease units; however, this study was conducted to evaluate the association between RA and comprehensive oral health status. Therefore, this study aimed to assess the association between RA and oral health using self-perceived oral health (SPOH) variables that can determine the oral health status in Korean adults using representative national data. Methods: Data from 40,186 selected participants were collected from the Korea National Health and Nutrition Examination Survey (KNHANES) between 2007 and 2018. The prevalence relative risk (PRR) was estimated using Poisson regression analysis to obtain the risk ratio of the SPOH according to RA. Results: The risk of SPOH depending on the RA status was statistically significant (odds ratio [OR] = 1.108, 95% confidence interval [CI] 1.005–1.222). In addition, the risk of SPOH depending on the RA status was higher in the group with diabetes mellitus (DM) (OR = 1.205, 95% CI 0.966–1.503) than in the group without DM (OR = 1.088, 95% CI 0.976–1.214). Conclusions: In this study, a significant association was identified between RA and SPOH. Oral health experts should identify the factors affecting the oral health of patients with RA and provide correct oral health care; however, additional research is needed.

## 1. Introduction

Health refers to a state of complete physical, mental, and social well-being. Health level is affected by both objective and subjective health status [1]. The objective health of an individual is assessed through clinical tests by medical personnel and can measure physical conditions such as illness or disability. Subjective health of an individual can predict more accurate levels of quality of life [2] and well-being and is considered an indicator of good health that reflects personal views on health that cannot be measured by medical methods [3]. When individuals rate their overall oral condition, it is referred to as self-perceived oral health (SPOH) and it can indicate one’s current oral health status. The perceived oral health status is related to health promotion; therefore, objective and subjective oral health must be considered [4].

Oral health is an essential part of an individual’s overall health and well-being and an important factor in digestion and nutrition. Improving oral health can affect the overall quality of life of individuals and society [5]. The World Health Organization [6] and World Dental Federation [7] emphasize that oral health is an essential component of overall health and quality of life and define it as a contributing factor to the physical, psychological, and social well-being of people [8].

Rheumatoid arthritis (RA) is a chronic disease that causes systemic inflammation, particularly of the joints, owing to adverse reactions of autoimmunity, and can impair daily activities [9]. Previous studies, which observed an association between RA and poor oral health, have shown similar mechanisms in the immune response between RA and periodontal disease (PD) [10]. Patients with PD were at a higher risk of developing RA [11] and patients with RA had fewer teeth [12]. The authors reported that periodontal bacterial DNA is a possible trigger factor for RA [13] and that patients with RA have a higher risk of PD than the general population [14,15,16]. Recently, the association between oral health-related quality of life and RA [17,18,19], a study evaluating oral health status and oral care reported by RA patients [20], and research reporting on oral hygiene and the dysfunction of temporomandibular joints (TMJ) in RA patients has been extensively studied [21,22]; however, this is the first study in northeast Asia. No studies in northeast Asia have focused on SPOH, an indicator of RA, and the comprehensive oral health status of an individual. Therefore, this study aimed to identify a potential relationship between RA and oral health by identifying SPOH in Korean adults using representative national data from the Korea National Health and Nutrition Examination Survey (KNHANES).

## 2. Methods

### 2.1. Study Design and Data Collection

#### 2.1.1. Study Design

Our study used data from KNHANES, which is a series of cross-sectional, nationally representative, population-based surveys on the health and nutritional status of Korean citizens conducted by the Korea Centers for Disease Control and Prevention [23].

#### 2.1.2. Inclusion and Exclusion Criteria

**Figure 1:**
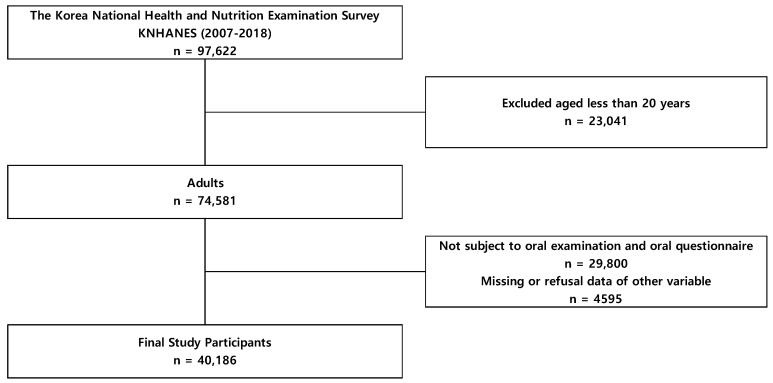
Schematic diagram of study participants.

Our study collected data from a total of 97,622 people who were surveyed between 2007 and 2018, representative of the Korean population. After excluding individuals aged under 20 years (*n* = 23,041), those who did not have an oral examination or complete the questionnaire (*n* = 29,800), and those who had missing data or data we could not use for other variables (*n* = 4595) (Figure 1), 40,186 people were selected as participants of this study.

### 2.2. Study Variables

#### 2.2.1. Exposure and Outcomes

The KNHANES includes a health interview, health examination, and nutrition survey. The interviews and check-ups were conducted by trained staff, such as doctors, medical technicians, and health interviewers.

As in previous studies, according to a self-administered questionnaire, “Have you been diagnosed with rheumatoid arthritis by a doctor?”, participants who answered “yes” to the diagnosis of RA [16] in the KNHANES health survey were defined as the RA group; pseudo-diagnosis was defined as the non-RA group.

SPOH was measured on a 5-point Likert scale (very good, good, normal, bad, very bad) in response to an individual’s perception of their oral health [24]. If a participant defined their oral health as “bad” or “very bad”, this was indicative of poor oral health.

#### 2.2.2. Other Variables

In this study, sex, age, income, and educational background were investigated in terms of demographic and social characteristics. Age was divided into four groups: 20–39 years, 40–59 years, 60–79 years, and 80 years of age or older. Income level was divided based on the median value, and the educational background of the participants was divided into two groups: high school or below and college or above.

According to previous studies, the prevalence of diabetes in patients with RA is high [25] and is also a primary risk factor for PD [26]; this makes diabetes a health status variable. This study was conducted on adults over the age of 20, and the diabetes model was viewed as common type 2 diabetes.

According to a study on oral health and disease activity in RA patients [27], RA patients who smoke had dry mouth, gingival bleeding, and difficulty chewing. Smoking is an important factor that negatively affects oral health [28] and is related to PD development in patients with RA [14,29]. Smoking experiences are divided into three groups according to the self-administered questionnaires: (i) Non-smokers mean those who have never smoked; (ii) ex-smokers mean those who smoked in the past but not currently; (iii) smokers mean those who currently smoke. In this study, non-smokers and ex-smokers were classified as “no” and current smokers were classified as “yes”. To determine oral health behavior, participants were asked how frequently they brushed their teeth each day. As in previous studies, responses were divided into ≥3 times a day and <3 times a day [30]. Participants were also asked whether they had attended an oral examination within the past year. Responses were divided into “yes” or “no”.

### 2.3. Statistical Analysis

Analysis of the data obtained from this cross-sectional study from the KNHANES (2007–2018) was conducted to identify an association between RA status and SPOH. For the general characteristics of the study subjects, frequency analysis, and descriptive statistics were conducted according to whether the participants had poor oral health. To obtain the risk ratio of the dependent variable according to the independent variable, the prevalence relative risk (PRR) was estimated using Poisson regression analysis [31]. In general, in cross-sectional studies, even if the prevalence odds ratio (OR) is obtained through logistic regression analysis, it is widely used to approximate the risk of prevalence comparison. However, the prevalence of focal health is not rare, therefore the risk of prevalence was calculated through Poisson analysis. To select an adjusted variable, a directed acyclic graph (DAG) was used [32] (Figure S1). Although it is known that RA may affect diabetes mellitus (DM) [33], some reports show that DM affects RA [34]; therefore, an additional DAG was implemented (Figure S2). As a result, sex, age, smoking, and DM were included as adjusted variables. Therefore, Model 2 defined sex, age, and smoking as adjusted variables, and Model 3 defined sex, age, smoking, and DM as adjusted variables. A complete denture may be observed as an outlier that is different from the general oral environment; therefore, sensitivity analysis was also performed, excluding individuals who had a complete denture. Data were analyzed using the SAS 9.4 software package (SAS Institute Inc., Cary, NC, USA).

## 3. Results

Table 1 lists the characteristics of the study participants according to whether they had poor oral health. RA was significantly higher in the group with poor oral health (52.6%, *p* < 0.0001). This group consisted mostly of men (44.9%, *p* < 0.0001), the elderly (51.6%, *p* < 0.0001), individuals with a low-income (49.4%, *p* < 0.0001) and a low level of education (47.6%, *p* < 0.0001), smokers (53.4%, *p* < 0.0001), individuals with DM (54.2%, *p* < 0.0001), and those who did not brush their teeth regularly (47.5%, *p* < 0.0001) and who did not have an oral examination within the last year (45.3%, *p* < 0.0001).

In this study, the number of women (80.1%, *p* < 0.0001), elderly (56.3%, *p* < 0.0001), individuals with lower income (57.7%, *p* < 0.0001) and education levels (87.9%, *p* < 0.0001), non-smokers (90.6%, *p* < 0.0001), individuals without DM (84.0%, *p* < 0.0001), and those who brushed their teeth less frequently (57.2%, *p* = 0.005) and did not attend an oral examination within the last year (75.9%, *p* = 0.0004) was higher in the RA group when compared to the non-RA group (Table S1).

As shown in Table 2, the crude OR of SPOH for RA in Model 1, considering the non-RA group as reference, was 1.214 (95% confidence interval [CI] 1.102–1.338). In Model 2, the OR for the RA group was 1.108 (95% CI 1.005–1.222), and the OR in Model 3 for the RA groups was 1.108 (95% CI: 1.005–1.222). All the age groups analyzed showed an increase in risk: 20–39 years (OR = 1.047, 95% CI 0.695–1.578), 40–59 years (OR = 1.211, 95% CI 1.015–1.443), and 60–79 (OR = 1.109, 95% CI 0.977–1.258); however, the risk decreased in the over 80 years group (OR = 0.871, 95% CI 0.52–1.457). The group with DM showed a higher risk than the group without DM (OR = 1.205, 95% CI 0.966–1.503). When sex, income, educational background, smoking, frequency of toothbrushing and oral examinations attended were stratified, most of the ORs did not show any considerable difference after the adjustment. These results are similar to those of the sensitivity analysis (Table S2).

## 4. Discussion

In this study, we identified an association between RA and SPOH. Previous studies have shown that patients with RA have fewer teeth [12], a higher reported risk of PD than the general population [14,15,16], and have a poor oral health quality of life [17,18,19] when compared to individuals without RA. The observations from these studies support the results from our current study.

RA is an autoimmune disease characterized by an accumulation of inflammatory cells that occurs due to changes in the systemic immune function. RA destroys cartilage and joint bone tissue and causes physical disabilities [35] as well as pain, swelling, and disability in TMJ [21,22]. RA and PD have a common biomedical mechanism [36] of bone destruction caused by inflammatory cytokines [35,37].

In general, patients with RA may have a reduced ability to perform oral hygiene due to the limited movement of the wrist and finger joints [35,38], thereby increasing inflammatory activity and the risk of developing PD. In addition, RA patients often had oral pain or discomfort due to poor periodontal conditions and had difficulty brushing their teeth [20]; gingival bleeding also occurred while brushing teeth and was frequently diagnosed with periodontitis [39]. If brushing teeth is difficult due to joint motion restrictions, it is recommended to improve oral hygiene by the use of electric toothbrushes [40].

Subgroup analysis stratified by age and diabetes status highlighted differences in the association between RA and oral health. There was an association between poor oral health and RA in the diabetic group. This observation was consistent with previous studies that reported poor oral health due to tooth decay or PD in adults with diabetes [41]. Therefore, the risk of poor oral health in RA patients with diabetes is increased. Subgroup analysis of those aged 80 years or older revealed a non-significant association between oral health and RA. The accumulated oral health status of the elderly is affected by various diseases. Previous studies report multiple tooth losses [42] and the development of systemic diseases in elderly patients [43]. This might explain the lack of association between RA and oral health in the elderly. Previous studies showed interactions in sex [44,45], smoking [27], and frequency of toothbrushing [18] stratification, but in this study, there was no considerable difference even after adjustment in the stratified subgroup analysis.

This study had several strengths. First, the number of samples used for analysis was sufficient and represented the entire nation. Second, to our knowledge, there has been no study in northeast Asia that can show a comprehensive oral health status using SPOH variables. This study is the first in northeast Asia to use SPOH, an index that comprehensively shows the oral health status of RA patients, for analysis.

This study has some limitations. First, it was difficult to reveal a causal relationship because the National Health and Nutrition Survey is cross-sectional. The cross-sectional data showed that the duration of the disease could not be defined and the diabetes model was considered a general type 2 diabetes, considering that it was an adult over the age of 20, so we think it would be difficult to represent the entire diabetes group. To prove a causal relationship, it is necessary to conduct a longitudinal study in the future. Second, correction variable data that were missing from the database remain unknown and could not be corrected. Despite these limitations, this study is valuable as it examines data from previous studies using the DAG method, includes them as adjusted variables, and analyzes them using representative national data.

We would like to make some suggestions for further research. First, we should find major causes such as demographic factors, health statuses like diabetes, and oral health behavior that threaten the oral health of RA patients and propose active information exchange and cooperation between rheumatologists and dentists to acquire the correct oral health status of RA patients. Second, we think preventive activities are necessary to achieve satisfactory oral health by inducing regular oral examinations of RA patients and developing oral care programs considerate of age.

## 5. Conclusions

In the presence of RA, poor oral health was statistically significant. There was an increase in the risk of poor oral health in the entire group with RA, especially in the diabetic group. Oral health experts should identify the factors affecting the oral health of patients with RA and provide correct oral health care; however, additional research is needed.

## Figures and Tables

**Table 1:** Characteristics of participants according to poor oral health.

	Poor Oral Health		
	No	Yes	*p*-Value
Total	22,687 (56.5)	17,499 (43.6)	
Sex			
Men	9553 (55.1)	7787 (44.9)	<0.0001
Women	13,134 (57.5)	9712 (42.5)	
Age			
20–39	7459 (64.5)	4115 (35.6)	<0.0001
40–59	8879 (57.1)	6681 (42.9)	
60–79	5888 (48.7)	6212 (51.3)	
≥80	461 (48.4)	491 (51.6)	
House Income			
Low	8846 (50.6)	8621 (49.4)	<0.0001
High	13,841 (60.9)	8878 (39.1)	
Education			
High school or below	15,151 (52.4)	13,745 (47.6)	<0.0001
College or above	7536 (66.8)	3754 (33.3)	
Smoking			
Non-/ex-smoker	19,144 (58.8)	13,442 (41.3)	<0.0001
Current smoker	3543 (46.6)	4057 (53.4)	
Diabetes mellitus			
No	20,585 (57.8)	15,008 (42.2)	<0.0001
Yes	2102 (45.8)	2491 (54.2)	
Tooth brushing			
<3	11,031 (52.5)	9967 (47.5)	<0.0001
≥3	11,656 (60.8)	7532 (39.3)	
Oral examination within 1 year			
No	15,433 (54.7)	12,800 (45.3)	<0.0001
Yes	7254 (60.7)	4699 (39.3)	
RA *			
No	22,311 (56.6)	17,081 (43.4)	<0.0001
Yes	376 (47.4)	418 (52.6)	

* Rheumatoid arthritis.

**Table 2:** Odds ratios of self-perceived oral health according to rheumatoid arthritis.

	Model1 ^†^	Model2 ^‡^	Model3 ^§^
Total	1.214 (1.102–1.338)	1.108 (1.005–1.222)	1.108 (1.005–1.222)
Sex			
Men	1.229 (0.995–1.518)	1.146 (0.928–1.416) ^a^	1.147 (0.928–1.418) ^b^
Women	1.232 (1.104–1.375)	1.1 (0.985–1.229) ^a^	1.1 (0.985–1.228) ^b^
Age			
20–39	1.044 (0.693–1.572)	1.047 (0.695–1.577)	1.047 (0.695–1.578)
40–59	1.177 (0.988–1.403)	1.208 (1.013–1.44)	1.211 (1.015–1.443)
60–79	1.107 (0.976–1.255)	1.11 (0.978–1.26)	1.109 (0.977–1.258)
≥80	0.878 (0.525–1.467)	0.865 (0.517–1.447)	0.871 (0.52–1.457)
House income			
Low	1.132 (0.999–1.282)	1.075 (0.948–1.218)	1.073 (0.946–1.216)
High	1.246 (1.067–1.455)	1.155 (0.988–1.35)	1.161 (0.993–1.356)
Education			
High school or below	1.161 (1.049–1.285)	1.113 (1.005–1.232)	1.112 (1.004–1.232)
College or above	1.066 (0.76–1.494)	1.009 (0.719–1.415)	1.013 (0.722–1.42)
Smoking			
No	1.258 (1.135–1.395)	1.113 (1.003–1.235) ^c^	1.113 (1.003–1.234) ^d^
Yes	1.176 (0.882–1.568)	1.053 (0.789–1.406) ^c^	1.053 (0.789–1.406) ^d^
Diabetes mellitus			
No	1.196 (1.073–1.332)	1.088 (0.976–1.214)	-
Yes	1.212 (0.974–1.509)	1.205 (0.966–1.503)	-
Tooth brushing			
<3	1.169 (1.031–1.325)	1.112 (0.98–1.262)	1.109 (0.978–1.259)
≥3	1.257 (1.078–1.465)	1.099 (0.941–1.282)	1.103 (0.945–1.287)
Oral examination within 1 year			
No	1.213 (1.087–1.353)	1.108 (0.992–1.237)	1.107 (0.991–1.236)
Yes	1.175 (0.952–1.451)	1.089 (0.881–1.347)	1.094 (0.885–1.352)

^†^ Crude odds ratio was calculated by Poisson analysis; ^‡^ Adjusted odds ratio was calculated by Poisson analysis after adjusting for sex, age, and smoking; ^§^ Adjusted odds ratio was calculated by Poisson analysis after adjusting for sex, age, smoking, and diabetes mellitus; ^a^ Adjusted for age, smoking; ^b^ Adjusted for age, smoking, and diabetes mellitus; ^c^ Adjusted for sex, age; ^d^ Adjusted for sex, age, and diabetes mellitus.

## Data Availability

Not applicable.

## Supplementary Materials

The following are available online at https://www.mdpi.com/article/10.3390/healthcare10030427/s1, Table S1: Characteristics of participants according to rheumatoid arthritis status, Table S2: Odds ratios of self-perceived oral health according to rheumatoid arthritis excluding the person with the complete dentures, Figure S1: Directed acyclic graphs for variable selection, Figure S2: Directed acyclic graphs for variable selection.
