# Supplementary material for: Association between Rheumatoid Arthritis and Poor Self-Perceived Oral Health in Korean Adults
Source: Healthcare (Basel). 2022 Feb 24;10(3):427. doi: 10.3390/healthcare10030427 (PMC8954365; doi:10.3390/healthcare10030427)
Supplement: Supplementary file 1 [file healthcare-10-00427-s001.zip › healthcare-1567298-supplementary.pdf]

Supplementary Materials

Table S1. Characteristics of participants according to rheumatoid arthritis status.

	Non-RA	RA *	*p*-Value
Total	39,392(100)	794(100)	
Sex			
Men	17,182(43.6)	158(19.9)	<0.0001
Women	22,210(56.4)	636(80.1)	
Age			
20–39	11,512(29.2)	62(7.81)	<0.0001
40–59	15,308(38.9)	252(31.7)	
60–79	11,653(29.6)	447(56.3)	
≥80	919(2.3)	33(4.2)	
House Income			
Low	17,009(43.2)	458(57.7)	<0.0001
High	22,383(56.8)	336(42.3)	
Education			
High school or below	28,198(71.6)	698(87.9)	<0.0001
College or above	11,194(28.4)	96(12.1)	
Smoking			
Non-/ex-smoker	31,867(80.9)	719(90.6)	<0.0001
Current smoker	7525(19.1)	75(9.5)	
Diabetes mellitus			
No	34,926(88.7)	667(84.0)	<0.0001
Yes	4466(11.3)	127(16.0)	
Tooth brushing			
<3	20,544(52.2)	454(57.2)	0.005
≥3	18,848(47.9)	340(42.8)	
Oral examination within 1 year			
No	27,630(70.1)	603(75.9)	0.0004
Yes	11,762(29.9)	191(24.1)	

* Rheumatoid arthritis.

Table S2. Odds ratios of self-perceived oral health according to rheumatoid arthritis excluding the person with the complete dentures.

	Model1 †	Model2 ‡	Model3 §
Total	1.213(1.1–1.337)	1.108(1.005–1.223)	1.108(1.004–1.223)
Sex			
Men	1.22(0.985–1.511)	1.139(0.92–1.411) [a]	1.14(0.92–1.412) [b]
Women	1.233(1.105–1.377)	1.103(0.987–1.232) [a]	1.102(0.987–1.232) [b]
Age			
20–39	1.044(0.693–1.573)	1.047(0.695–1.578)	1.048(0.695–1.579)
40–59	1.175(0.985–1.401)	1.205(1.01–1.35)	1.207(1.012–1.44)
60–79	1.104(0.973–1.254)	1.109(0.976–1.26)	1.107(0.974–1.258)
≥80	0.914(0.546–1.529)	0.903(0.54–1.512)	0.907(0.542–1.519)
House income			
Low	1.129(0.996–1.281)	1.073(0.945–1.218)	1.071(0.944–1.216)
High	1.249(1.07–1.458)	1.158(0.991–1.354)	1.164(0.996–1.36)
Education			

High school or below	1.16(1.048–1.285)	1.113(1.004–1.234)	1.113(1.004–1.233)
College or above	1.067(0.761–1.495)	1.009(0.719–1.415)	1.013(0.722–1.421)
Smoking			
No	1.258(1.134–1.395)	1.114(1.003–1.236)[c]	1.113(1.003–1.236)[d]
Yes	1.17(0.875–1.564)	1.049(0.783–1.405)[c]	1.048(0.783–1.404)[d]
Diabetes mellitus			
No	1.195(1.072–1.332)	1.089(0.976–1.215)	-
Yes	1.211(0.97–1.511)	1.206(0.965–1.509)	-
Tooth brushing			
<3	1.166(1.027–1.323)	1.111(0.978–1.262)	1.108(0.975–1.258)
≥3	1.26(1.081–1.469)	1.102(0.944–1.286)	1.106(0.948–1.291)
Oral examination within 1 year			
No	1.211(1.085–1.352)	1.108(0.992–1.238)	1.107(0.99–1.237)
Yes	1.178(0.954–1.454)	1.091(0.883–1.349)	1.095(0.886–1.354)

† Crude odds ratio was calculated by Poisson analysis; ‡ Adjusted odds ratio was calculated by Poisson analysis after adjusting for sex, age and smoking; § Adjusted odds ratio was calculated by Poisson analysis after adjusting for sex, age, smoking and diabetes mellitus; [a] Adjusted for age, smoking; [b] Adjusted for age, smoking and diabetes mellitus; [c] Adjusted for sex, age; [d] Adjusted for sex, age and diabetes mellitus.

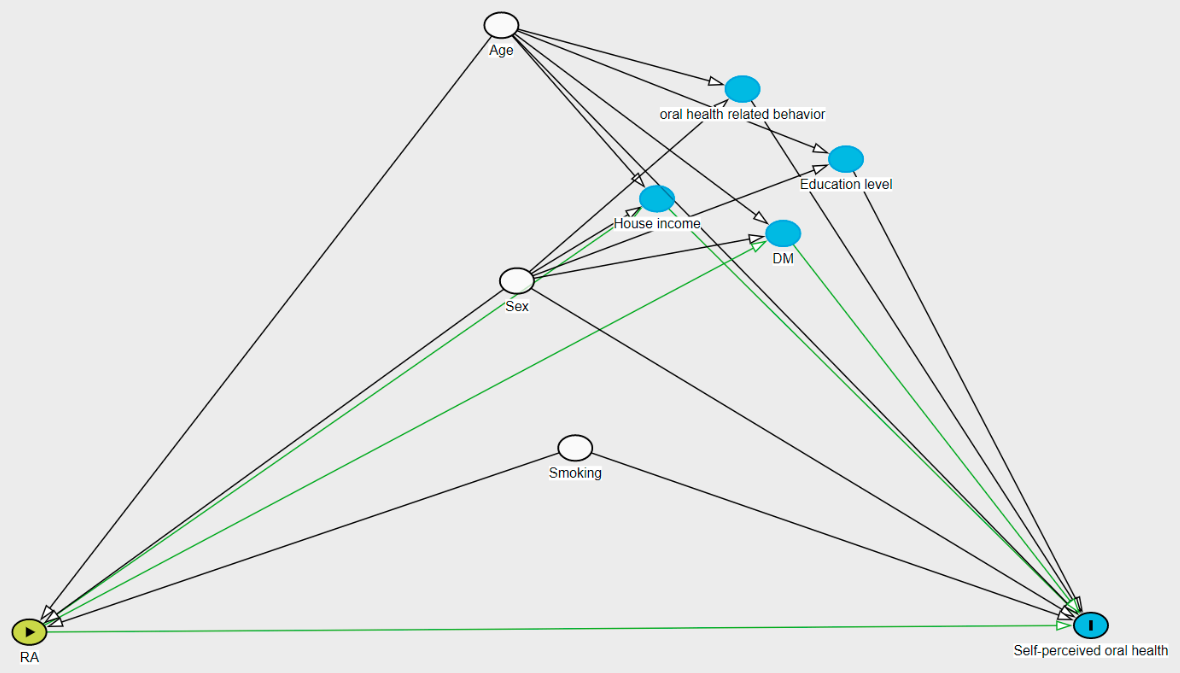

Figure S1. Directed acyclic graphs for variable selection.

Directed acyclic graphs (DAGs) to show the hypothesized causal associations among rheumatoid arthritis (RA) (yellow-green circle), self-perceived oral health ("I" in a blue circle), and covariates. The proposed adjustment variable using the DAGitty is indicated by white circles. Ancestor variables of the outcome and not proposed adjustment variables are indicated by blue circles.

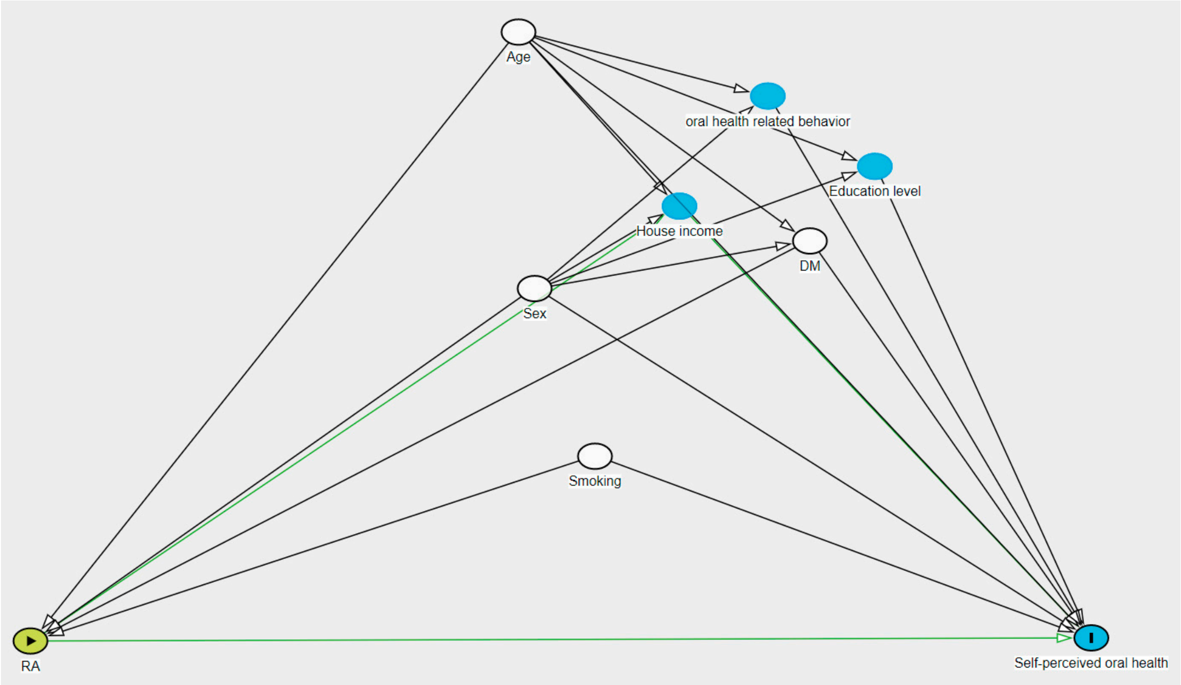

Figure S2. Directed acyclic graphs for variable selection.

Directed acyclic graphs (DAGs) to show the hypothesized causal associations among rheumatoid arthritis (RA) (yellow-green circle), self-perceived oral health (“I” in a blue circle), and covariates. The proposed adjustment variable using the DAGitty is indicated by white circles. Ancestor variables of the outcome and not proposed adjustment variables are indicated by blue circles.
